# Behaviour reactions of bottlenose dolphins (*Tursiops truncatus*) to multirotor Unmanned Aerial Vehicles (UAVs)

**DOI:** 10.1038/s41598-019-44976-9

**Published:** 2019-06-12

**Authors:** Ticiana Fettermann, Lorenzo Fiori, Martin Bader, Ashray Doshi, Dan Breen, Karen A. Stockin, Barbara Bollard

**Affiliations:** 1 New Zealand Institute of Applied Ecology, School of Science, Auckland University of Technology, 46 Wakefield, WU Building, 1010 Auckland, New Zealand; 2 Coastal-Marine Research Group, Institute of Natural and Mathematical Sciences, Massey University, Private Bag 102 904, North Shore, MSC New Zealand

**Keywords:** Animal behaviour, Behavioural ecology

## Abstract

Unmanned aerial vehicles (UAVs) represent a novel and cost effective research tool to investigate cetacean behaviour, as conventional aircraft are expensive, limited in the altitude they can fly at and potentially disturb sensitive wildlife. In addition, the aerial observation from the UAVs allows assessment of cetacean behaviour from an advantageous perspective and can collect high spatial and temporal resolution data, providing the opportunity to gather accurate data about group size, age class and subsurface behaviour. However, concerns have been raised about the potential risks of disturbance to animals caused by the UAV’s visual and acoustic stimuli. Boat-based surveys were conducted to assess the short-term behavioural responses of resting bottlenose dolphins (*Tursiops truncatus*) to a lightweight Vertical take-off and landing (VTOL) UAV flown at 10, 25, and 40 m altitude. Changes in group swim direction and frequencies of surface and aerial behavioural events were recorded from an anchored research vessel before (control) and during the aerial survey. The number of reorientation and tail slap events increased significantly between controls and flights when the UAV was flown at 10 m over the animals. In contrast, no significant differences were detected when the aircraft was flown at 25 and 40 m altitude. However, a precautionary approach is recommended for research applications requiring lower flight altitudes, with further research recommended to assess how different cetacean species and age class may respond to the UAV presence.

## Introduction

UAVs are providing a safe method for scientists to acquire high-resolution remote sensing data at lower cost and increased operational flexibility^1^ and it is rapidly becoming a common practice both for marine mammal researchers and whale-watchers^2^. To date, UAVs have been successfully used for a number of marine mammal research applications (for a review see^2^). Nevertheless, United States government agencies have been systematically using Remotely Piloted Aircraft (RPA) to conduct several marine mammal surveys since 2014^3^. In particular, it has been demonstrated that VTOL UAVs are effective and efficient tools for pinniped colony counts^4,5^ and cetacean photogrammetry^6–8^. VTOL UAVs have been also used to collect samples of whale’s exhaled breath condensate^9^ with their application in cetacean behavioural studies now under investigation^2^. In comparison with boat-based surveys, multirotor aircraft can assess cetacean behaviour from a more advantageous perspective^10^ as most cetacean activities take place below the surface, out of sight of boat-based observers. Furthermore, the presence and noise of a research vessel may affect cetacean behavioural responses and bias observations^11,12^. However, there is also a potential risk of disturbance to wildlife by the UAV^13–16^. It is well documented that the noise produced by conventional aircraft and helicopters elicit strong behavioural responses in cetaceans^17–20^. In contrast, research on the impacts of UAVs on cetaceans is limited to opportunistic observations, and most studies do not quantify behavioural responses^21^. Quantifying disturbance levels is not straightforward as several factors including species^20^, ecotype^22^, behavioural state^20^, environmental factors^21^ and the noise levels of the aircraft itself can influence responses to the aircraft presence. With the recent increase in research, commercial and recreational UAV operations around cetaceans^23^, researchers and regulatory bodies urgently need baseline data to develop guidelines and avoid animal harassment^13,14,21^.

In the absence of previously studies in the peer reviewed literature dedicated to the UAV disturbance assessment, this study is the first to specifically assess UAV disturbance levels on the behaviour of a cetacean species. We measured the short-term behavioural responses by sampling behavioural events of free-ranging bottlenose dolphins (*Tursiops truncatus*) near Great Barrier Island, New Zealand to a VTOL UAV flying 10 m, 25 m and 40 m overhead. We considered the number of group reorientation events as indicative of responses to the aircraft. Additionally, we quantified surface behaviour events that can potentially represent stress responses (tail and chin slaps)^24^ and visual interest (spy hop and side float)^25,26^ towards the UAV. This can have a significant impact on the population, where the UAV noise can disturb and interrupt biologically significant behaviours (*i*.*e*. resting) which may carry energetic costs and affect individual fitness. Short-term effects can have potential long term population consequences^27,28^.

Our tests focussed on resting groups, and these events were interpreted as indicative of the animals moving from resting behaviour to more active behaviours. We monitored behavioural events from an anchored vessel with the engine off^29–31^ for a control period without UAV and during UAV flights. We also investigated whether environmental factors played a role in the observed response.

## Methods

### Study site and species

The study was conducted between July 2015 and December 2016 at Great Barrier Island (GBI; 36°10′S, 175°23′E), New Zealand. The island is situated 90 km northeast of Auckland City (36°51′S, 174°46′E) within the outer Hauraki Gulf and covers an area of 285 km^2^. The predominantly rocky shoreline is characterized by several sheltered bays and inlets^32^. The research site included the inshore waters of the western side of the island between Miners Head and Ross Bay. This region has been identified as a potential hotspot for bottlenose dolphins, with dolphins observed year-round, exhibiting evidence of site fidelity^33,34^. Most of the area is uninhabited and marine mammals are not targeted by commercial marine mammal tour operators. Research was conducted following the permission granted by the Maritime New Zealand Safe Ship Management system for commercial vessels and by New Zealand Department of Conservation (DOC) for UAV operations over marine mammals.

### Marine mammal survey methodology

Non-systematic surveys were conducted on board a research vessel (Osprey 8.5 m, dual Honda four stroke 150 hp). Once a group of bottlenose dolphins was sighted, the vessel approached in accordance with the New Zealand Marine Mammal Protection Regulations (1992). The boat moved at idle speed to minimise effects on dolphins’ behaviour^12,35^. At 300 m from the group of dolphins, time and GPS location were recorded, as well as environmental parameters (weather, Beaufort Sea State, Douglas Sea Scale and water depth). Initial behavioural data and group size were recorded, with group defined as any number of dolphins observed in association, moving in the same direction and engaged in the same behaviour^36^. Group size was estimated based on a minimum visual count or estimate of animals observed after scanning the group. For each group, the number of individuals was recorded in categories to the nearest five animals (1–5, 6–10, 11–15 etc.).

### UAV operations

**Figure 1:**
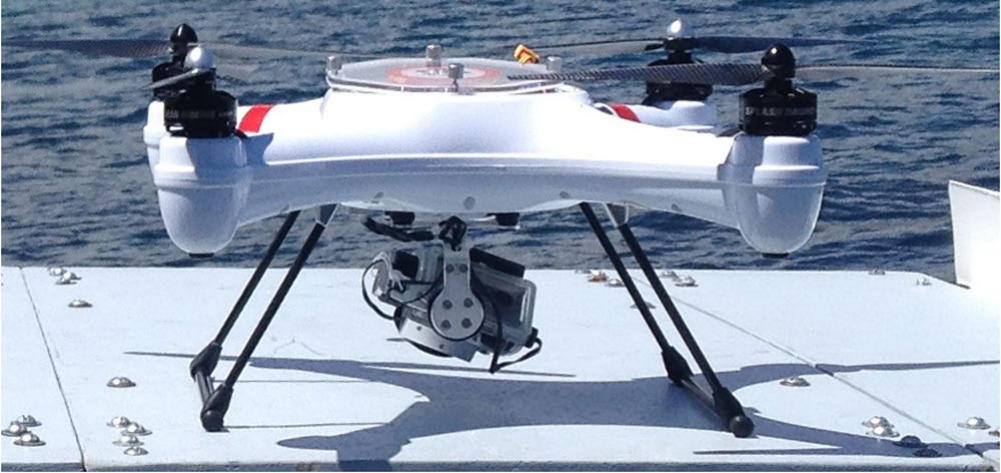
Splashdrone (SwellPro, Shenzhen, China) on custom built foldable helipad on research vessel, *AUT Sciences*.

UAV operations were conducted under marine mammal research permit 499890-MAR issued by New Zealand Department of Conservation (DOC) and complied with New Zealand Civil Aviation Authority (CAA) regulations. The UAV used was the SwellPro Splashdrone (Shenzhen, China) (Fig. 1), a waterproof four bladed helicopter (diagonal diameter of 550 mm, 2.3 kg, carbon fibre propellers and produce a mean of 95 dB re 1 μPa root mean square (rms) of noise level, www.swellpro.com). The Splashdrone was fitted with DJI Naza M-V2 flight controller (Shenzhen, China) and was equipped with a Hero4 GoPro video camera attached to a gimbal. The UAV can be controlled from up to 1 km away in open areas and has a flight endurance of 12 minutes with 70% consumption of its’ 5200 mAh LiPo battery. The UAV was launched from the anchored research vessel at a minimum distance of 100 m from the dolphins, in accordance with the DOC permit. The vessel sat at anchor for 30 mins before flying commenced to allow dolphins to habituate to the presence of the vessel and any responses to engine noise to subside. From the take off point, the UAV climbed vertically to the randomly predetermined height (10 m, 25 m and 40 m) and was then manoeuvred horizontally towards the group at the same altitude for 10 minutes.Thirty-minute breaks between each test was taken to allow any responses of the animals to the UAV to subside. The aerial surveys were conducted in locations with similar geomorphological characteristics (sandy bays within a maximum depths of 15 m) to reduce the number of factors to be considered in the analysis.

### Data collection

Before and while the UAV was flown above the dolphins, surface behavioural (Table 1) and group reorientation events were recorded by the primary observer (Ticiana Fettermann). Reorientation was considered to have occurred when there were changes of group swim direction of 90° or more with respect to the heading direction.

**Table 1:** Definition of surface behaviour events of bottlenose dolphins.

Side float	Dolphin floats on the surface of the water on its side so that the flipper is visible and one eye is clear of the water.
Spy hop	Dolphin rises with its head vertically above the water surface so that both the eyes are clear of the water.
Tail slap	Dolphin strikes the surface of the water with its tail.
Chin slap	Dolphin strikes the surface of the water with its rostrum.

When evident, the observer annotated whether a surface event was repeated multiple times by the same individual. The predominant behaviour state was assessed every one-minute via scan sampling method^37^ to ensure the group was resting throughout the tests. Resting behaviour was defined when more than 50% of the animals were observed in a tight group, moving slowly in a constant direction^30,35,38,39^. All dolphins were scanned from left-to-right to ensure inclusion of all individuals and avoid potential biases caused by specific individuals and/or behaviours^35^. Data were collected for 10 minutes prior to UAV launch (control), and during the 10 minutes exposure to aircraft (impact) from an anchored research vessel. To assess the UAV disturbance level for bottlenose dolphin, the aerial videos were discarded, and only boat-based data collected (control and impact) were used to compare number of behavioural events.

### Statistical methods

Statistical analysis were conducted using the statistical analysis and graphics software R version 3.4.3 (R Development Core Team, 2017).

A generalized linear mixed effects model (GLMM) with negative binomial error distribution and log link (glmmADMB)^40^ was generated for the number of reorientation events. The main effects of presence/absence of the UAV at different altitudes (UAVALT), time of the day (TOD), Beaufort Sea State (BSS), weather (W, sunny vs. cloudy), group size (GS) and the interaction between BSS and UAVALT. To account for the repeated observations on the same group, we included group identity as a random term. Potential collinearity issues were assessed using generalized variance inflation factors (GVIF = VIF^[1/(2*df)]^), which were compared to their collinearity thresholds (maximum VIF value of 10^[1/(2*df)]^, which translated into 1.26 for UAVALT and 3.16 for the remaining predictors) (R package car)^41^. All GVIFs were below their collinearity thresholds.

GLMMs with a binomial distribution and logit link (R package *lme4*)^42^ were generated for the side-floats, tail-slaps, spy-hops and chin slaps. A two-column matrix holding the number of successes (number of animals in a group exhibiting behavioural events) and failures (number of animals per group not showing a behavioural action) was provided as response variable^43^. These binomial GLMMs contained UAVALT, TOD, BSS, W and the interaction between BSS and UAVALT. The tail-slap, spy-hop and chin-slap models showed inflated standard errors of the parameter estimates, suggesting separation issues. Remodeling those variables using a Bayesian GLMM with a weak prior resolved the separation issues (R package *blme*)^44^. Assessing the significance of the explanatory variables followed a backwards selection based on AIC^45^. Post-hoc analyses were performed using a multiple comparison procedure based on Tukey contrasts (R package *lsmeans*)^46^. The Benjamini and Hochberg^47^ method was used to adjust *P*-values for multiple testing (R package *multcomp*)^48^.

## Results

Free-ranging bottlenose dolphins were exposed to a lightweight VTOL UAV (SwellPro *Splashdrone*) flying for ten minutes at a fixed altitude over the animals in resting behavioural state. Twenty-five flights were conducted on seven independent groups resting in sheltered bays off the South-West side of Great Barrier Island, New Zealand. All UAV operations took place in light wind conditions (max wind speed of 10 knots and Beaufort Sea State 1–2). The hypothesis was that dolphins would respond to the UAV at different altitudes by changing the frequencies of reorientation and surface behavioural events during the flight (impact). A total of 23 UAV flights at the altitude of 10 m (N = 7), 25 m (N = 7) and 40 m (N = 9) were analysed. Two additional flights at 10 m were further discarded from the analysis as dolphins changed behaviour and were subsequently displaced from the area before completion of the impact flight.

**Figure 2:**
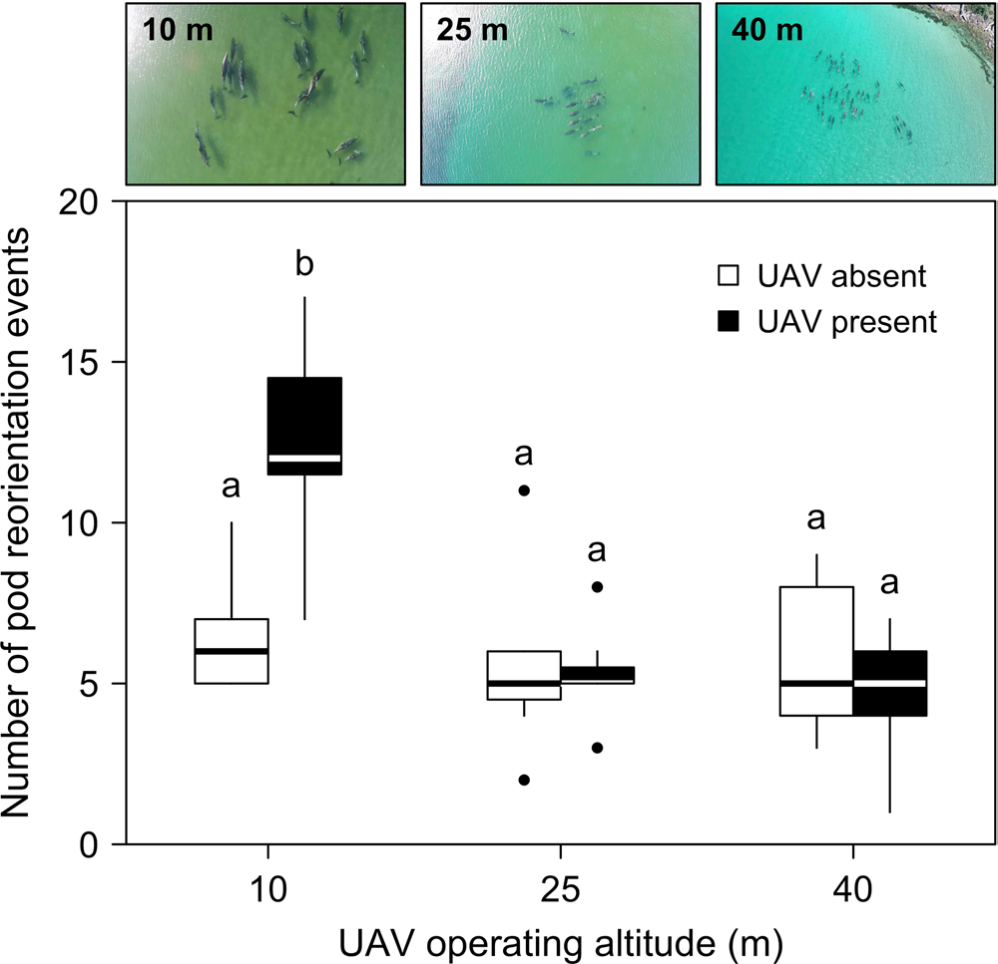
Number of pod reorientation events as a function of unmanned aerial vehicle (UAV) absence or presence at 10, 25, and 40 m operating altitude. Different lower-case letters indicate statistically significant differences at *α* = 0.05 (multiple comparison procedure using Tukey contrasts). Black filled circles indicate outliers (first quartile − 1.58 × interquartile range or third quartile + 1.58 × interquartile range). Bottlenose dolphins were photographed during UAV disturbance tests around Great Barrier Island, New Zealand (36°10′S, 175°23′E).

**Figure 3:**
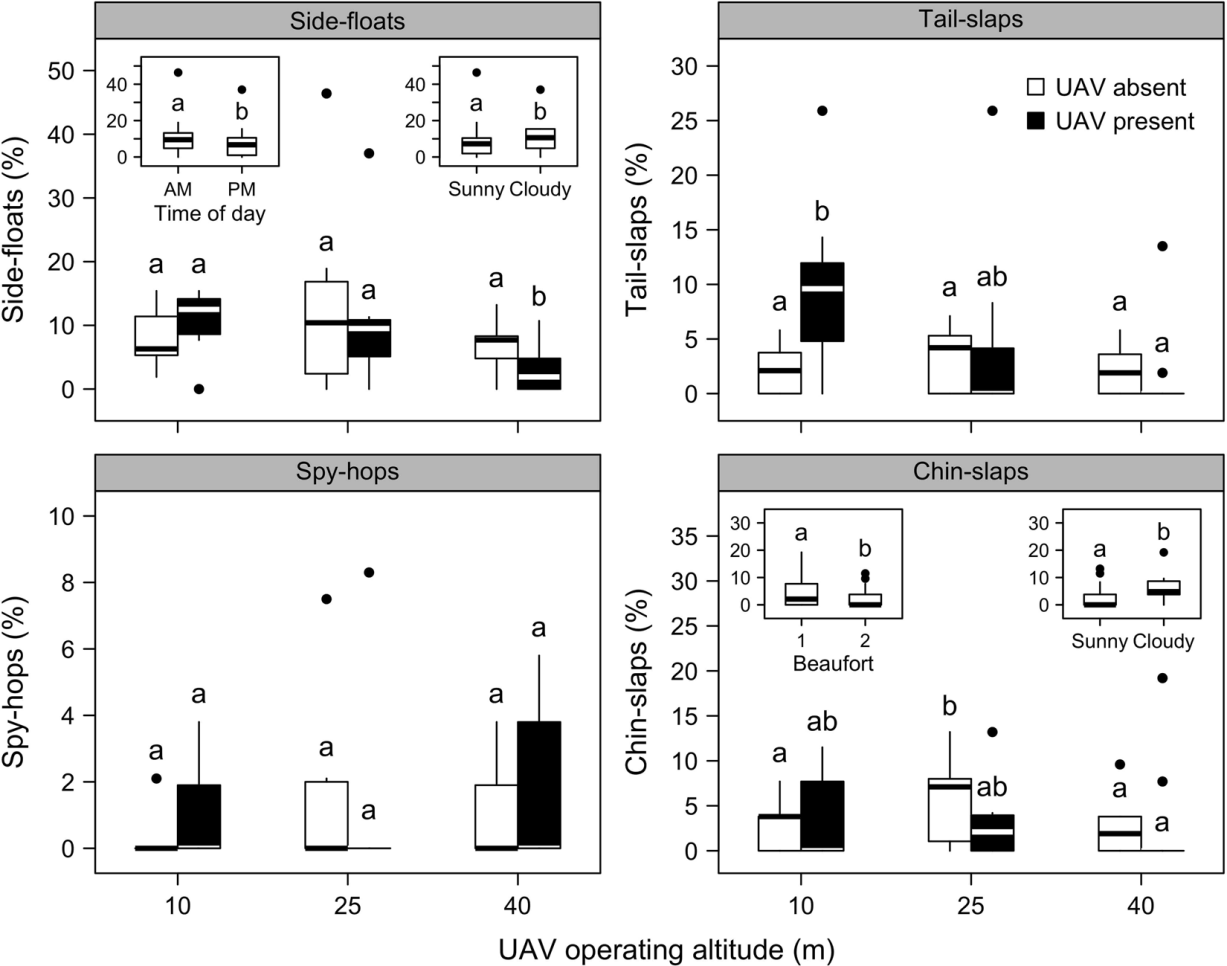
Behavioural responses of bottlenose dolphins (*Tursiops truncatus*) to the presence of an unmanned aerial vehicle (UAV) at 10, 25 and 40 m operating altitude. The behavioural events are expressed as the proportion of animals in groups showing this type of behaviour (*n* = 5 groups). Inset plots share the same *y*-axis title with the surrounding plot and show additional statistically significant predictors, if applicable. Different lower-case letters indicate statistically significant differences at *α* = 0.05 (multiple comparison procedure using Tukey contrasts; insets: generalized linear mixed effects model output). Black filled circles indicate outliers (first quartile − 1.58 × interquartile range or third quartile + 1.58 × interquartile range).

The results on the lowest AIC model show that flight altitude had a significant effect on reorientation events. Two-fold increase in group reorientation events was observed when operating at 10 m of altitude, but no significant effect at higher operation altitudes (Fig. 2 and Table 2). In contrast, the individual-based behavioural responses remained largely unaffected by the presence of the UAV regardless of the operating altitude, apart from the tail-slaps which showed a 4.5-fold increase in response to the UAV flying at 10 m altitude (Fig. 3 and Table 2). Side-floats were statistically more frequent in the morning and on cloudy days (Fig. 3 insets, Table 2). Chin-slaps occurred more often when the Beaufort seas state was 1 compared to state 2, and they were also observed more frequently on overcast compared to sunny days (Fig. 3 insets, Table 2).

**Table 2:** Backward selections performed on generalized linear mixed effects models (GLMM) for bottlenose dolphin behavioural events.

Model	AIC	*L*	*df*	*P*
***Reorientation events***				
UAVALT + TOD + BSS + W + PS + UAVALT × BSS	229.5			
UAVALT + BSS + W + GS + UAVALT × BSS	229.6	2.06	1	0.151
UAVALT + TOD + BSS + GS + UAVALT × BSS	227.8	0.22	1	0.639
UAVALT + TOD + BSS + W + UAVALT × BSS	227.8	0.24	1	0.624
UAVALT + TOD + BSS + W + GS	220.5	0.98	5	0.964
UAVALT + BSS	217.7			
BSS	242.9	35.2	5	<0.001***
**UAVALT**	**216**.**1**	0.42	1	0.518
***Side-floats***				
UAVALT + TOD + BSS + W + UAVALT × BSS	229.7			
UAVALT + BSS + W + UAVALT × BSS	237.6	9.88	1	0.002**
UAVALT + TOD + BSS + UAVALT × BSS	237.1	9.36	1	0.002**
UAVALT + TOD + BSS + W	229.1	9.33	5	0.097
TOD + BSS + W	238.6	19.50	5	0.002**
UAVALT + BSS + W	244.3	17.30	1	<0.001***
UAVALT + TOD + BSS	238.1	11.06	1	<0.001***
**UAVALT** + **TOD** + **W**	**228**.**6**	1.55	1	0.212
TOD + W	239.3	20.65	5	<0.001***
UAVALT + W	242.5	15.87	1	<0.001***
UAVALT + TOD	236.5	9.88	1	0.002**
***Tail-slaps***				
UAVALT + TOD + BSS + W + UAVALT × BSS	172.8			
UAVALT + BSS + W + UAVALT × BSS	173.0	2.19	1	0.139
UAVALT + TOD + BSS + UAVALT × BSS	174.1	3.31	1	0.069
UAVALT + TOD + BSS + W	172.8	9.97	5	0.076
UAVALT + BSS	178.5			
BSS	190.4	21.87	5	<0.001***
**UAVALT**	**176**.**6**	0.13	1	0.721
***Spy-hops***				
UAVALT + TOD + BSS + W + UAVALT × BSS	107.9			
UAVALT + BSS + W + UAVALT × BSS	109.8	3.91	1	0.048*
UAVALT + TOD + BSS + UAVALT × BSS	106.6	0.73	1	0.394
UAVALT + TOD + BSS + W	104.8	6.91	5	0.228
UAVALT + TOD + BSS	103.7			
UAVALT + BSS	104.8	3.15	1	0.076
UAVALT + TOD	103.0	1.31	1	0.253
TOD + BSS	98.0	4.37	5	0.497
***Chin-slaps***				
UAVALT + TOD + BSS + W + UAVALT × BSS	157.9			
UAVALT + BSS + W + UAVALT × BSS	156.8	0.93	1	0.335
UAVALT + TOD + BSS + UAVALT × BSS	162.3	6.76	1	0.009**
UAVALT + TOD + BSS + W	156.3	8.43	5	0.134
**UAVALT** + **BSS** + **W**	**155**.**1**			
UAVALT + BSS	162.9	9.82	1	0.002**
UAVALT + W	160.9	7.79	1	0.005**
BSS + W	162.5	17.36	5	0.004**

The first column shows the fixed term of the GLMMs (UAVALT = combined factor of UAV absence/presence and operating altitude, TOD = time of day, BSS = Beaufort Sea State, W = weather, GS = group size). Bold fixed terms indicate the best GLMM specification as judged by the AIC and likelihood ratio tests. AIC = Akaike Information Criterion, *L* = likelihood ratio statistic, *df* = degrees of freedom, *P* = *P*-value for the comparison between full and reduced models. Grey cells indicate the full models of each round of the backwards selection process. Blank cells (*L*, *df* and *P* columns) are associated with the original full model or a newly structured full model resulting from previous model comparisons. Note that for the spy-hops data none of the tested explanatory variables was statistically significant at the end of the backwards selection.

## Discussion

We quantified the short-term behavioural responses of bottlenose dolphins to a lightweight VTOL UAV (*Splashdrone*) flying at three different altitudes. Our results show that flying at 10 m elicits a quantifiable response of resting animals. That is, the number of group reorientation and tail slaps events increased between controls and flights (Figs 2 and 3). In contrast, we observed that flying the UAV at 25 m or higher had no significant effect on dolphin’s behaviour (Figs 2 and 3).

Surface behavioural events (e.g., tail slap, side float, spy hop, chin slap) and swimming patterns (e.g., bearing consistency, dive time) are used by researchers to quantify short term responses of delphinids to either acoustic or visual disturbance sources^49^. For example, the increase in the numbers of directional changes can underlie horizontal avoidance and has been reported for bottlenose dolphin^29,30,50,51^ and killer whales (*Orcinus orca*)^52^ exposed to powerboats. The increase in frequency of aggressive behaviours, such as tail slap and chin slap, can represent a response to disturbance^24^, whereas side floating and spy hopping might indicate an attempt to visualize the noise source^25,36^. The results presented in this study suggest that bottlenose dolphins noted and reacted to the aircraft flying at 10 m altitude, increasing the number of reorientation and tail slaps events.

Noise produced by manned aircraft flying at low altitude elicit strong behavioural responses in several species of cetaceans^17,19,20^. While the literature detailing potential disturbance caused by UAVs on cetaceans is scarce^21^, recent studies documented that pinnipeds can change their behaviour in the presence of lightweight VTOL RPAs flying at 30 m and below^15,53^. Meanwhile, Durban, *et al*.^7^ did not observe evidence of behavioural responses in killer whales (*Orcinus orca*) exposed to a VTOL UAV flying at 35 m altitude. Several species of baleen whales and sperm whales (*Physeter microcephalus*) have been approached by VTOL UAV flying lower than 10 m altitude showed no apparent reaction^9^. However, these prior observations were not focussed on the detection of behavioural responses, and were designed to experimentally assess and quantify the responses levels of animals to different flight altitudes. Moreover, it is important to highlight that different types of UAVs may produce different noise levels, depending for example on their propulsion system, electric motors, propellers, weight, speed and many other factors^23^. Consequently, the potential effect of UAVs on marine mammals will depend on the study species and the behavioural context of the animal at the time of flying^21^.

**Figure 4:**
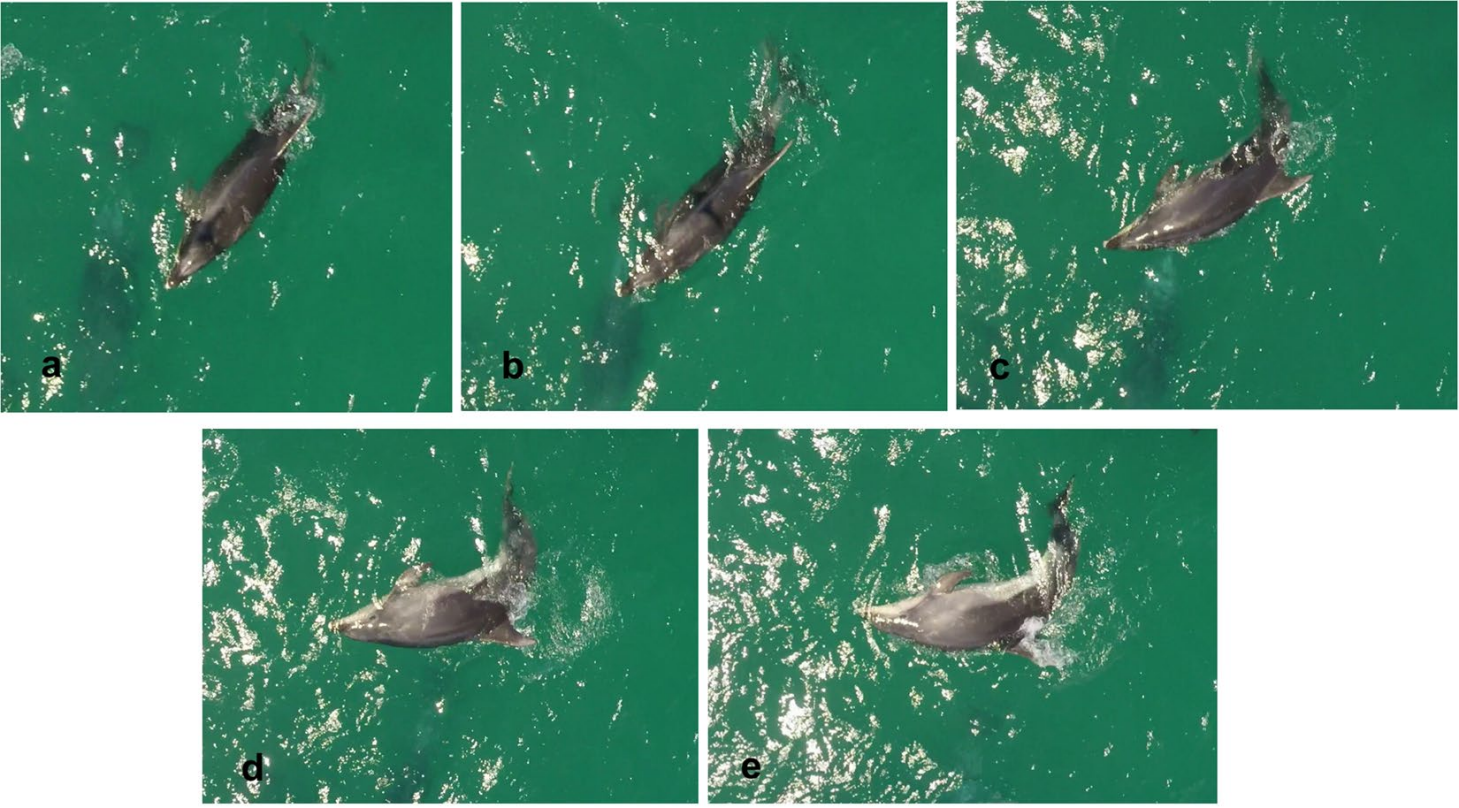
Side float sequence cropped from the aerial video captured by the UAV at 10 m of altitude. Note: the shadow of the UAV on top of his head (**a**) just before performing side float (**b**–**e**).

The ‘Splashdrone’ flying at 10 m produces noise levels between 91 and 97 dB re μPa rms [mean of 95 dB re 1 μPa (rms)] at 1 m depth^23^. It is believed that odontocetes like bottlenose dolphins are able to hear this acoustic stimuli, although Christiansen, *et al*.^23^ suggests that the effect is likely to be small, even when the animals are close to the surface. However, bottlenose dolphins, like other marine mammals are highly active at the surface, and are able to hear airborne noise^24^. Therefore, it is important to also considerer atmospheric noise levels (@ 1 m of 80 dB re 20 μPa), as they are significantly higher that underwater levels^23^. Furthermore, bottlenose dolphins are documented as avoiding the shade of a helium-filled tethered balloon used for aerial surveys^54^. The UAV in this study is considerably smaller in size than the balloon, and casts a smaller shadow. However, one dolphin was observed to perform a side float just after the aircraft shadow cast past over his head when flying at 10 m altitude. This event could have been in response to the UAV shadow cast over its body, though this cannot be confirmed (Fig. 4).

As discussed prior, some cetacean species apparently react strongly to aircraft, while others appear less affected. Nevertheless, an experimental assessment of cetacean behavioural responses to UAVs is not always practicable and there may be many confounding variables. The potential effect of UAVs on marine mammals may depend on the behavioural state of the animals at the time, as well as environmental factors (sea state, wind speed and geomorphology) and the presence and type of other anthropogenic activities^21^. While environmental factors can modulate the acoustic stimuli received by cetacea^55^, our survey design aimed to minimize the potential effects of independent variables. The UAV was flown in a maximum wind speed of 10 knots with a maximum Beaufort Sea State of 2 over only resting animals in similar habitats. This relationship is quite important, as it is more likely that the UAV noise will travel more when the sea is calmer with windless conditions than when there are ripples, whitecaps and wind. Experiments occurred only when no other human activities nearby occurred, while the research vessel maintained a minimum distance of 100 m away with its engines off.

UAVs offer distinct advantages in the sampling of marine mammals. For example, the creation of a high quality video that can be reviewed multiples times, offers improved assessments by reducing interpretational bias. Indeed, UAVs provide the opportunity to gather accurate data about group size, age class, and has a great potential to collect behavioural data from an advantageous perspective in a non-invasive way. However, results presented here still suggest that UAV similar to the one used in this study should conservatively only be flown at over 10 m for bottlenose dolphins. It was evident that dolphins react towards the UAV when flying at 10 m, even with a limited sample size. Unfortunately, our sample size was relatively small, we were only able to investigate resting behaviour and as the UAV was frequently unable to fly due the poor weather and sea conditions. future research, therefore, should further identify the threshold at which disturbance occurs (i.e. between 10 and 25 m) and also identify how this differs during different behavioural states other than resting. It is also important to investigate how dolphins are likely to respond to different UAV angle and height approaches. During Vas *et al*.^16^ study, birds showed behavioural reactions when exposed to UAV approaching vertically, but no reactions when approached horizontally. Unfortunately, during our study we only approached the dolphins horizontally during all the flights, to maximize our flight time and data collection. Furthermore, the size and type of the UAV platform when approaching wildlife to conduct behavioural observations should also be considered, due to the fact that some species may be more sensitive to UAV noise, presence and/ or shadow than others.

The findings presented here strengthen the argument that further research on the potential impact of UAVs on wildlife^13,16^ and marine mammals^2,21^ is required to avoid the risk of harassment. Precautionary research approaches are preferred, with and the assessment of disturbance levels recommended to be conducted during the early conceptual stages of study design^14^. Finally, knowledge gained from disturbance assessments will provide invaluable guidance for the regulation of recreational and commercial use of UAVs around wildlife.
