# Author Correction: Behaviour reactions of bottlenose dolphins (*Tursiops truncatus*) to multirotor Unmanned Aerial Vehicles (UAVs)

**DOI:** 10.1038/s41598-020-73527-w

**Published:** 2020-10-08

**Authors:** Ticiana Fettermann, Lorenzo Fiori, Martin Bader, Ashray Doshi, Dan Breen, Karen A. Stockin, Barbara Bollard

**Affiliations:** 1 New Zealand Institute of Applied Ecology, School of Science, Auckland University of Technology, 46 Wakefield, WU Building, 1010 Auckland, New Zealand; 2 Coastal-Marine Research Group, Institute of Natural and Mathematical Sciences, Massey University, Private Bag 102 904, North Shore, MSC New Zealand

Correction to: *Scientific Reports* 10.1038/s41598-019-44976-9, published online 12 June 2019

This Article contains an error. The following sentence in the Introduction,

“In the absence of previously studies in the peer reviewed literature dedicated to the UAV disturbance assessment, this study is the first to specifically assess UAV disturbance levels on the behaviour of a cetacean species.”

should read:

“With limited previous work^1^ in the peer reviewed literature dedicated to the UAV disturbance assessment, this study is among the first to specifically assess UAV disturbance levels on the behaviour of a cetacean species.”
